# Using Downscaled GRACE Mascon Data to Assess Total Water Storage in Mississippi Alluvial Plain Aquifer

**DOI:** 10.3390/s23146428

**Published:** 2023-07-15

**Authors:** Zahra Ghaffari, Greg Easson, Lance D. Yarbrough, Abdel Rahman Awawdeh, Md Nasrat Jahan, Anupiya Ellepola

**Affiliations:** 1 Department of Geology & Geological Engineering, University of Mississippi, University, MS 38677, USA; geasson@olemiss.edu (G.E.); ldyarbro@olemiss.edu (L.D.Y.); mjahan@go.olemiss.edu (M.N.J.); avellepo@go.olemiss.edu (A.E.); 2 Mississippi Mineral Resources Institute, University of Mississippi, University, MS 38677, USA; 3 Department of Civil & Environmental Engineering, University of Mississippi, University, MS 38677, USA; amawawde@go.olemiss.edu

**Keywords:** GRACE mascon, downscaling, total water storage, random forest model

## Abstract

The importance of high-resolution and continuous hydrologic data for monitoring and predicting water levels is crucial for sustainable water management. Monitoring Total Water Storage (TWS) over large areas by using satellite images such as Gravity Recovery and Climate Experiment (GRACE) data with coarse resolution (1°) is acceptable. However, using coarse satellite images for monitoring TWS and changes over a small area is challenging. In this study, we used the Random Forest model (RFM) to spatially downscale the GRACE mascon image of April 2020 from 0.5° to ~5 km. We initially used eight different physical and hydrological parameters in the model and finally used the four most significant of them for the final output. We executed the RFM for Mississippi Alluvial Plain. The validating data R^2^ for each model was 0.88. Large R^2^ and small RMSE and MAE are indicative of a good fit and accurate predictions by RFM. The result of this research aligns with the reported water depletion in the central Mississippi Delta area. Therefore, by using the Random Forest model and appropriate parameters as input of the model, we can downscale the GRACE mascon image to provide a more beneficial result that can be used for activities such as groundwater management at a sub-county-level scale in the Mississippi Delta.

## 1. Introduction

Groundwater management is critical for community sustainability across the world and interest in groundwater storage and its changes is growing globally. Groundwater resources are the most important resources of water for human use, irrigation, and industry [1,2]. At the same time, groundwater depletion is becoming more wildly recognized as a severe worldwide issue that threatens water sustainability and critical ecosystems [3].

According to [2], water storage changes could happen due to different factors including: (a) climate (arid vs. humid) and climate extremes (droughts and floods), (b) human intervention through water use (irrigation mostly), (c) source of water use (surface water, groundwater), and (d) surface reservoir management. Climate regulates hydrologic systems with groundwater discharging to surface water in humid regions, while surface water frequently replenishes groundwater in dry regions [2].

The stress on the groundwater supplies in the Southeast of the United States (US) is related to a variety of reasons such as population growth, climate change, urbanization, agricultural expansion, and drought which could negatively impact the water resources [4]. During the last 30 years, the southeast of the US has undergone multiple severe droughts, the most recent of which cost more than USD 1 billion in agricultural losses during 2006–2008 [5]. Drought has been studied extensively over the central and southwestern US in terms of causes and impacts. However, due to the region’s humid coastal environment, complicated climate, and its relative abundance of water, the drivers and mechanisms that contribute to drought are poorly understood in the southeast US [5]. Between 1900 and 2008, the volume of groundwater storage in the subsurface in the US declined by almost 1000 km^3^. Top three aquifers with the most water storage depletion are the High Plains aquifer of the Midwest, the Mississippi Embayment section of the Gulf Coastal Plain aquifer system, and the Central Valley of California [3].

The world’s greatest accessible source of fresh water is groundwater and in the United States, 63% of freshwater is used for irrigation purposes [1,2]. In the United States, approximately 40% of the water supply is derived from groundwater resources, and more than 40% of irrigation water depends on groundwater resources [6]. Groundwater is more reliable for continuous irrigation purposes compared to precipitation and surface water. The increased usage of groundwater for irrigation purposes has resulted in groundwater depletion in many places across the US [7]. Ref. [7] shows that groundwater recharge has a close relationship with irrigation plans and management practices over surface hydrology in agricultural watersheds in the state of Mississippi.

There are large depressions in the potentiometric-surface map in the Delta region in the Mississippi Alluvial Plain (MAP) [8]. The largest depression in the potentiometric surface is in the central part of the Delta region (west of Leflore County and east of Sunflower County), and the flow of water in the Delta region is toward this large depression. MAP region is a critical region in producing rice, catfish, and cotton nationwide. Heavy groundwater extraction and the use of surface water for irrigation has led to this depression [9].

High-resolution and continuous hydrological products are critical for improving the capacity of sustainable water resources management and predicting water-related patterns under climate change and human impacts [1]. The Gravity Recovery and Climate Experiment (GRACE) (2002–2018) and GRACE-FO (Follow-on) (2018–present) have successfully provided data on five major components of the hydrologic cycle on and below the surface of Earth: polar ice, soil moisture, surface and groundwater storage, and ocean mass distribution for the past 20 years with about monthly intervals. GRACE track Earth’s mass movements and changes, mainly in water, which then is converted to Total Water Storage Change (TWSC) that represents the stored water mass anomalies on and below Earth’s surface. The gravitational differences of even a 300-km disk of water with only one-centimeter thickness could be detected by the two GRACE satellites [10].

GRACE products have been extensively used for studying groundwater storage changes during these past years, and it provides valuable data support for studying groundwater variations. GRACE data may be particularly useful in assessing the long-term viability of future water management schemes aimed at resolving geographical and temporal disconnections between water supply and demand [2]. However, the coarse spatial resolution of GRACE products (originally ~300 km), creates a challenge for use in local-scale studies.

Researchers have used different methods to downscale GRACE data to make it possible to use them for local-scale hydrological investigations [1,11,12,13,14,15]. Generally, there are two common approaches to downscale satellites images: dynamical and statistical. Dynamical approaches need a greater resolution of images as input. In addition, these methods require extensive computation time and resources that might not be accessible by all users. Given the importance of selecting a proper hydrological model, some of these methods lack surface water or groundwater components [11,12,16]. In statistical downscaling approaches, the relationship between large-scale and small-scale variables are used to provide the final higher-resolution result [11,12].

Machine learning methods have achieved unprecedented performance thresholds in water science and related fields. Among all machine learning methods, Random Forest (RF), Artificial Neural Network (ANN), and Boosted Regression Tree (BRT) are the most used methods in downscaling GRACE images. For example, ref. [14] uses RFM and ANN approaches to downscale the GRACE data from 1° to 0.25° (nominally 110 km to 28 km) by using nine different inputs for the model (DEM; slope angle; slope aspect; soil moisture; evapotranspiration; rainfall; surface runoff; canopy water; and temperature). They aim to downscale GRACE products for the Indus Basin irrigation system for local water resource management use. Their validation results reveal that R^2^ between downscaled groundwater storage and observational wells groundwater storage are 0.67 and 0.77 at seasonal and annual scales with a confidence level of 95%, respectively. They conclude the RFM has the potential to downscale GRACE data at a spatial scale suitable to predict groundwater storage at regional scales. Ref. [17] used the BRT model to downscale GRACE images from 1° to 5 km for the karstic upper Floridan aquifer, by using precipitation, Land Surface Temperature (LST), NDVI, soil moisture anomaly, lithology, transmissivity, TWS, GWL as inputs. They had satisfying results (R^2^ = 0.79, NSE = 0.61) after evaluating spatial predictions with the 29 existing groundwater wells. They could demonstrate that the water level was stable from 2002 to 2016 in the area but it varied seasonally. Ref. [1] introduces a very fine resolution output (1 km) for China by using DEM, LST, precipitation, slope, NDVI, gross domestic product, Landsat tree cover continuous fields, land use status database, population, clay content, silt content, and soil content inputs in RF and XGBoost models. XGBoost is an ensemble learning technique that is based on a gradient-boosting decision tree algorithm. They validate their result by comparing the water level measurements of 251 in situ wells. The resulting R^2^ range from 0.77–0.89 for XGBoost and 0.74–0.86 for RF for 2004–2016. They also emphasize the reliability of their downscaled model by comparing the consistency of the interannual changes of nine river basins in China by using the images before and after downscaling. Another piece of research was conducted by [13] for downscaling GRACE from 1° to 0.25° spatial resolution by using an RF model. Their purpose is to provide a higher spatial resolution of groundwater storage for regional water management. They use precipitation, evapotranspiration, runoff, soil moisture, snow water equivalent, and canopy water to conduct their research from the 2006–2015 time period. Their verified result demonstrates a correlation between the downscaling results and the observation wells is 0.78 and 0.94, on the monthly scale and annual scale respectively. Both refs. [12,15] use an ANN model to generate final images with a spatial resolution of 16 km^2^. Ref. [12] used soil type, slope, annual precipitation, and temperature from 2002–2010 in California’s Central Valley. They develop an empirical model capable of downscaling GRACE to a high-resolution dataset for groundwater storage changes in their study area. In their study, the ANN model could effectively simulate groundwater storage change with acceptable Nash-Sutcliffe efficiency (NSE) values for calibration and validation (ranging from 0.2445 to 0.9577 and 0.0391 to 0.7511, respectively).

**Figure 1:**
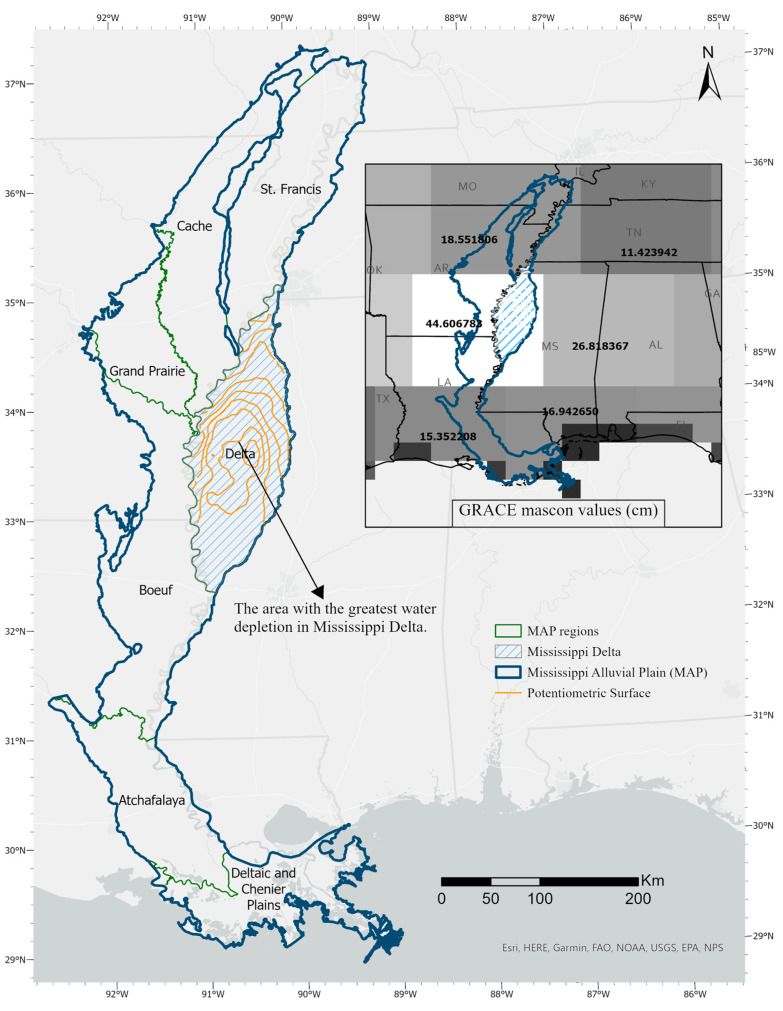
Location of Mississippi Alluvial Plain and the Mississippi Delta region within it.

The MAP located in the south-central United States is the focus of this study. The objective of this study is to assess the accuracy of GRACE mascon products in capturing the water level over a relatively small area such as the Mississippi Delta by downscaling the GRACE mascon images using a random forest model. Unlike other research that has used a time series of GRACE images and used different models for downscaling the images, we focus on one image from April 2020 and use specifically RFM to downscale the GRACE mascon image. According to [8], there is a large depression in Sunflower and Leflore counties in the Mississippi Delta region in 2020 as shown in Figure 1. We aim to find out if we can see this depression in the GRACE mascon downscaled image for 2020 or not. The final output has a ~5-km spatial resolution which makes it suitable for county-level and regional management activities in most affected groundwater areas in the Mississippi River Valley Alluvial. The easily accessible inputs (e.g., soil type, aquifer thickness, DEM, evapotranspiration, temperature, precipitation, NDVI, and land cover) makes this approach a potential method for downscaling GRACE mascon images in areas with similar aquifer and weather characteristics.

## 2. Methods and Materials

### 2.1. Study Area

The study area is a majority of the Mississippi River Alluvial Plain (MRAP) geophysical providence. The larger area of study is the Mississippi Alluvial Plain (MAP) which consists of the historic flood plain of the Mississippi River from Cairo, Illinois then south to the outfall of the river into the Gulf of Mexico (Figure 1). The Mississippi Delta is a focused subset of the study area and is a subregion of the MAP and contains the lower Mississippi River alluvial aquifer (LMRAA) known as Mississippi Delta. The Mississippi Delta is an important surficial aquifer and is located in the northwest of Mississippi with an area of 18,100 km^2^. LMRAA is a shallow alluvial aquifer in northwest Mississippi and the recharge remains through seasonal precipitation and could be quick.

This area is covered with dense agricultural activities and the irrigation water is mostly (98%) supplied from groundwater withdrawn from the alluvial aquifer [8,18,19,20]. MAP is a humid region but because it receives most of its rainfall outside of the growing season, groundwater and surface water are the main sources of water for irrigation purposes.

Situated in the northern portion of the MAP from approximately Vicksburg, Mississippi then north through the valley is underlain by the Mississippi River Valley Aquifer (MRVA). This aquifer is one of the heaviest pumped groundwater systems in the United States [21] and is connected to surface-water features in some regions and unconnected in others at least during part of the year, given spring 2020 groundwater and surface water measurements [8,22,23]. The alluvial aquifer is composed of Quaternary-age sands and gravel deposited after Wisconsin glaciation, making it an ideal surficial aquifer with many high-yield wells [18,20]. Although the MRVA is geologically a confined aquifer, it behaves as an unconfined aquifer regarding replenishing its water supply where surface water may or may not be hydrologically connected to the aquifer. This means that it is replenished directly from surface water (rivers and precipitation infiltration) with different recharge rate which depends on the precipitation characteristics, topography, vegetation coverage, depth to groundwater, and soil conditions [22]. In some areas of the aquifer, it exhibits confined or semiconfined conditions at least during part of the year.

While there is more than 1300 mm of annual rainfall in the area, not all areas could absorb precipitation since the alluvial aquifer is covered with a 10–20 m thick impermeable silt-clay layer over most parts of the Mississippi Delta. Because of this, improving aquifer management for a sustainable agriculture in the region is crucial [22]. Despite this amount of rainfall each year in the Mississippi Delta, and the existence of the Mississippi River in the area, the groundwater resources are under stress since more than 90% of its supply is being used for irrigation purposes which have resulted in significant areas of water level decline in parts of the aquifer. In terms of the volume of pumped water for irrigation purposes, the Ogallala aquifer of the High Plains region is the most productive agricultural aquifer in the U.S. followed by LMRAA and California’s Central Valley aquifers as the second productive agricultural aquifers [8,22,23]. According to [23] in 2005, 11 billion gallons of groundwater was pumped from the aquifers in the Mississippi embayment. Ref. [24] reports of 9.3 million acre-ft/year of groundwater plumage in California’s Central Valley aquifer.

### 2.2. Data

**Figure 2:**
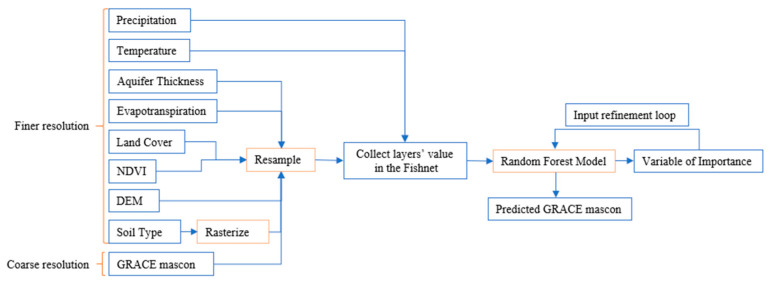
Process flow of the research.

We obtained eight different data layers as inputs and a mascon layer with an original resolution to use in the RFM. The data type and their spatial resolution are listed in Table 1 and a brief description about each layer is provided below. we made an effort to obtain all data to align with the month of the GRACE mascon data which is April 2020. The approach for downscaling is shown in Figure 2.

#### 2.2.1. Total Water Storage

GRACE measures changes in Earth’s gravity field at monthly intervals, which are caused by redistributions of mass on the planet. These redistributions can be static, such as the location of continents and mountains, or time-varying, such as oceanic and atmospheric circulations and changes in terrestrial water storage. The range and range-rate measurements from GRACE’s two satellites are used to create a map of these temporal variations in the gravity field [25]. Filtering is crucial for GRACE data processing. However, it is important to note that this step can introduce signal leakage and attenuation, which inevitably impacts the accuracy and quality of global and regional mass change estimates. There are two types of GRACE signal leakage, that is, leakage-in and leakage-out. The presence of a “leakage-in” error refers to the phenomenon where signals from the surrounding area inadvertently influence the measurements in the target area of interest. On the other hand, the “leakage-out” error describes the situation where signals from the target area of interest unintentionally impact the measurements in the surrounding area [26]. The measurements are pre-processed to remove the effects of atmospheric pressure and high-frequency oceanic motion, leaving anomalies in terrestrial water storage. These anomalies are then analyzed using either spherical harmonics or the mass concentration (MASCONs) [27] approach to produce monthly gravity solutions. In contrast to unconstrained spherical harmonic solutions, the constrained mascon solutions derived from geophysical models offer the advantage of not requiring destriping or smoothing processes. Additionally, these mascon solutions exhibit reduced susceptibility to leakage errors compared to harmonic solutions [10]. The errors in the spherical harmonics solutions, tend to be smaller in larger regions and larger in smaller regions. Specifically, the errors can be as small as 1–2 cm in equivalent height of water in continental-scale river basins, but can be large enough to overwhelm the hydrology signal in regions smaller than approximately 150,000 km^2^ [28,29] so as an alternative, mass concentrations (MASCONS) is used.

The primary advantage of using the mass concentration approach is that each mascon has a specific known location. This allows for the incorporation of prior information (constraints) into the data inversion process, which can help to remove correlated errors in the gravity solution without the need for destriping or smoothing. The mascon approach also allows for a better separation of land and ocean signals [25]. Additionally, ref. [30] evaluated the level-3 mascon solutions, which are a type of data product generated using the mass concentration approach, and found that they did not suffer from leakage problems that were present in earlier mascon levels. This makes level-3 mascons a suitable choice for use in our downscaling model. GRACE mascons solutions are available from various centers, including the Jet Propulsion Laboratory in California [31].

#### 2.2.2. Temperature and Precipitation

We obtained precipitation and mean temperature data from Parameter-elevation Regressions on Independent Slopes Model (PRISM) dataset for the month of April 2020 [32]. This data is available daily and monthly at ~4 km spatial resolution for the U.S. Dataset values are stored in the standard metric units used for climatology, precipitation uses millimeters and temperature are in units of degrees Celsius.

#### 2.2.3. DEM

Elevation was captured from Mississippi Automated Resource Information System (MARIS) [33] data portal, where statewide 10 m DEM were available to download.

#### 2.2.4. Aquifer Thickness

We obtained the MRVA thickness data at a 1-km resolution image from [34]. These were produced from the efforts of the USGS Water Availability and Use Science Program (WAUSP). The program was charged with a multiyear task to assess groundwater availability and other water resources in the MAP. The data are an interpolated surface using the extensive borehole data of the region.

#### 2.2.5. Soil Type

We obtained soil type data from USDA Natural Resources Conservation Service [35]. These data are in vector format which we converted to raster with the same resolution as PRISM data using a majority filter.

#### 2.2.6. Normalized Difference Vegetation Index

We used NASA’s Moderate Resolution Imaging Spectroradiometer (MODIS) to retrieve data for the Normalized Difference Vegetation Index (NDVI) of the study area for 14 April 2020. This dataset provides information about vegetation growth and health across the Earth’s surface and provides important information for monitoring changes in vegetation over time. Aqua MODIS sensor was used for the MODIS NDVI V6 collection. The satellite overpassed daily for the 16-day interval over the study area and processed data product at 1 km resolution [36].

#### 2.2.7. Land Cover

MODIS MCD12Q1 V6 product with yearly intervals was used for 2020. This product provides information about the type and extent of land cover across the Earth’s surface and has six different classification schemes with a spatial resolution of 500 m. Supervised classification of MODIS Terra and Aqua reflectance data were used to generate this product. From the data, we chose Land Cover Type 5 (annual plant functional types [PFT]) classification has 11 classes [37]. We preferred to use the PFT classification since it includes “cereal croplands” as one of the classes which is the dominant land cover in the Delta region out of five different classes of land cover that exist in the Delta region. There are eight different classes in the MAP region and the “cereal croplands” and “deciduous broadleaf trees” are the two dominant classes in the area.

#### 2.2.8. Evapotranspiration

The evapotranspiration data were downloaded from the EarthExplorer portal on 22 April 2020. The Aqua MODIS MYD16A2GF Version 6.1 Evapotranspiration/Latent Heat Flux (ET/LE) product is a 500-m pixel resolution image with 8-day temporal resolution and is used in this study [38].

#### 2.2.9. Ground-Based Measurement

The Yazoo Mississippi Delta Joint Water Management District (YMD) is tasked with recording and maintaining groundwater data throughout the Mississippi Delta by measuring depth to groundwater within the unconfined alluvial aquifer twice annually (early April and October). There are approximately 700 wells continuously monitored for the past 20 years.

### 2.3. Methods

#### 2.3.1. Random Forest Approach

The spatial downscaling method is based on the relationship between GRACE mascon and various environmental variables. Random forest is a decision tree-based, non-parametric, and ensembled approach based on classification and regression tree which uses randomization when selecting the features at each node [13,39,40]. Ref. [41] explains that random forest models combine both the distribution of all the decision trees in a given forest and the independently sampled random vector for the predictor. The tree predictor is based on the classification and regression trees (CART) algorithm [40]. Random forest uses equal weights when selecting training samples. This method estimates each input parameter’s contribution to the model by recognizing any reduction in predictive performance while randomly altering a predictor parameter [39]. The RF algorithm could be outlined as follows:

1. Random Subsets: For each tree, a random subset of the training data is selected with replacement (bootstrapping).
2. Tree Construction: At each node, a random subset of features are selected to determine the split.
3. Predictions for new samples: each tree in the random forest makes a prediction. The final output prediction can be made by averaging the predictions from all the individual regression trees:

(1) $$f=\frac{1}{N}\sum_{t=1}^{N}f_{i}\left(x\right),$$

where *N* is the number of trees and *f_i_*(*x*) is the prediction from each individual regression tree [40].

The main advantage of Random Forest is that it reduces overfitting by using multiple trees and randomly selecting subsets of features and data for each tree. It also has the ability to handle both numerical and categorical data and can provide information on feature importance. For this study, we use the Random Forest classification tool from ArcGIS Pro version 3.0.0. This tool is made with the same basic concepts of the Random Forest technique put forward by [41]. The default was selected in the tool and it matched with parameters.

#### 2.3.2. Data Pre-Processing

After collecting all data, we projected them to the correct projected coordinate system. Then we clipped each raster layer to the MAP border. Following, we resampled them to the same size as PRISM data (~5 km). We used Nearest Neighbor resampling method to resample GRACE mascon, and the Cubic Convulsion method to resample NDVI, evapotranspiration, land cover, DEM, and aquifer thickness. After rasterizing soil type data, we used the majority method to resample it. We created a fishnet to cover the whole study area with the same size as other raster data. Then by using spatial join, we collected the value of each cell of the input layers in the fishnet vector layer. Therefore, we created a vector layer (called Fishnet now) that has recorded the value of each input layer for every single cell inside the study area to use in the model. Appendix A is an example of the first 10 rows of the Fishnet. We used this vector data as the input in the “Forest-based Classification and Regression” tool in ArcGIS Pro and run the RFM. We accepted the default in the RF model in ArcGIS Pro and used 100 trees and put 10% of the data aside for validation purposes. After running the model, it returned the predicted GRACE mascon as a vector layer and a “Variables of Importance” (VI) table. We first executed the model using all eight layers of input. After running the model, considering the importance of each input by using the VI table, we excluded all layers that had less than 10% importance (including NDVI, evapotranspiration, land cover, and soil type). For defining this threshold, we considered 1σ which is equal to 0.095. Six types of soil exist in the MAP region and have a total of 0.03 importance. The eight types of land cover in the region had even a lower VI 0.01 importance. Therefore, the final results are based on using four inputs of the greatest VI including mean temperature, precipitation, aquifer thickness, and elevation (Table 2).

For the model assessment purposes, we have the water level from 272 wells in the Delta, MS, area for April 2020 which is provided for us by the Yazoo Mississippi Delta Join Water Management District (YMD). Since the water level data exist only for the Delta region (not the entire MAP region), we only focused on the assessment for the Delta region. There are only 263 cells out of 916 cells in the Delta area that have well(s) in them, we created the predicted groundwater level surface of the Delta area by using the spatial Kriging tool in ArcGIS Pro to interpolate a surface from the well data.

## 3. Results

**Figure 3:**
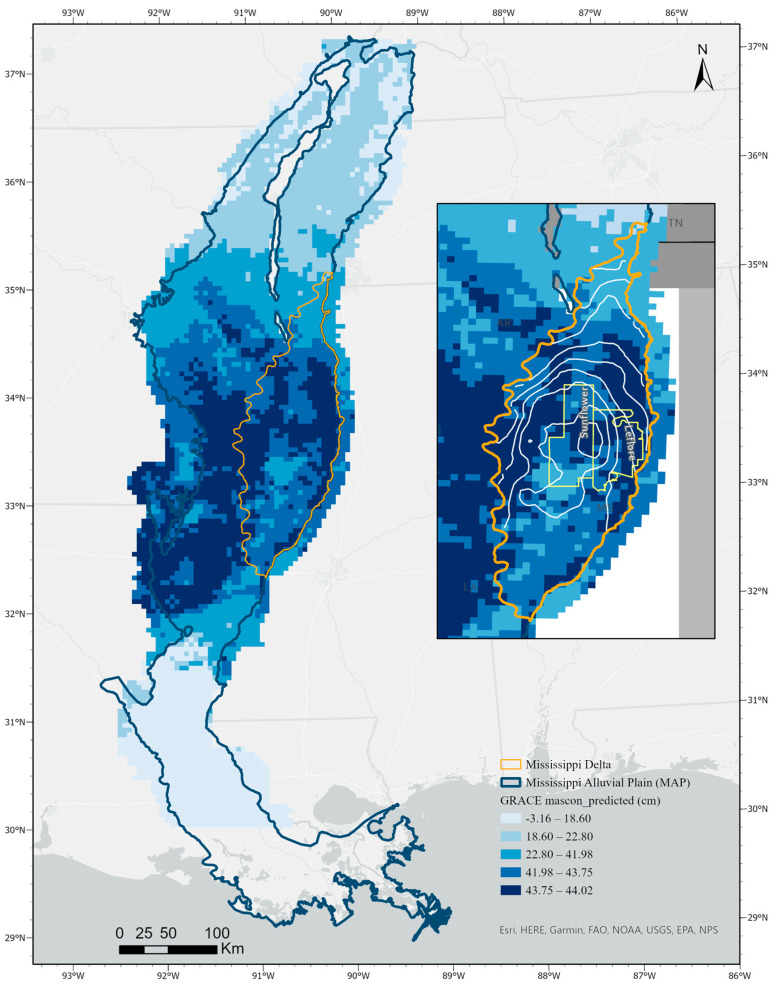
Downscaled GRACE Mascon TWS for MAP region for April 2020.

Table 2 is the table of the variable of importance which ranked the importance of each used input for the MAP region. As it is shown in this table, the mean temperature and precipitation have the highest importance with the value of 0.35 and 0.33, respectively, followed by elevation and aquifer thickness with the importance value of 0.15 and 0.12 respectively. Therefore, we ran the model using four important inputs and the model output is illustrated in Figure 3. In the model, the soil type and land cover were treated as categorical data, so the importance of each class is calculated separately which is less than 1%.

The output of the forest-based classification and regression tool for the MAP region is shown in Figure 3. We used quantile classification in ArcGIS Pro to better illustrate the predicted GRACE mascon values in five different classes. The result (Figure 3) shows that less water mass exists in southeast Arkansas where more rice fields exist, northeast Louisiana, and the central Delta region where the water depletion is reported.

We used R^2^, RMSE, and MAE statistical metrics to evaluate the model-predicted output. The R^2^ closer to 1, RMSE and MAE closer to 0 indicate a better model. The forest-based classification and regression model reports two R^2^ for the output. The first is a correlation coefficient of the comparison of the training data subset between the predicted and observed values. The second good fit metric is a comparison using the entire dataset between the predicted and observed values. Table 3 displays the statistical metrics for the model for the MAP region. Illustrated in Table 3, the large R^2^ both for training and validating data, 0.88 and 0.85, respectively, and low RMSE and MAE is indicative of high accuracy in the predicted GRACE mascon values.

The wells’ water level data is available only for the Delta region, therefore, we created contours of water level similar to the USGS 2020 potentiometric surface report to compare our results with. There are 272 wells located in 263 cells out of 916 cells in the Delta region. After creating the interpolated groundwater surface layer by using the Kriging tool and creating the contour layer, we can confirm that the water depletion reported by [8] was observed in our data trends as well (Figure 3).

## 4. Discussion

The temperature and precipitation show the highest importance in predicting the GRACE mascon value. Our finding is similar to [14] which showed that precipitation is the most important variable in predicting the mascon value followed by the temperature. Ref. [14] used Pearson’s correlation coefficient between all nine variables to determine the correlation of each independent variable with the TWS before running the model. They found that rain-fall, soil moisture, surface runoff, and canopy water storage have the strongest correlation, and temperature showed the lowest correlation with TWS. Despite their findings, they used all nine independent variables in the model to predict the TWS. After running the model and having the result of it, they run the Variable Importance Measures Predictive (VIMP) to check the predictability of independent variables by the model which revealed that rainfall was the crucial variable in the RF model. Soil moisture and temperature were the second most influential variables in training the RFM to predict the TWS despite the fact that temperature did not show a high correlation with TWS.

Since mascon is reporting the water level changes for each month, and the results (Figure 3) is showing smaller mascon values in the central toward the south of the Delta region, we can say that there is less water mass above the baseline. This result aligns with the report from [8] which said that the largest depression in the potentiometric surface has happened in the central part of the Delta region. This area is crucial in agricultural production in Mississippi [9]. Ref. [19] has reported the association between groundwater withdrawals and streamflow depletion in the Sunflower River as well.

Ref. [20] uses Generalized Additive Model (GAM) to look at the interaction between groundwater and surface water in five sites throughout the Mississippi Delta for the month of April of each year. They find significant declines in groundwater level (8–12 m decrease) between 10 April 1980 and 10 April 2016 in the Big Sunflower site in the Sunflower area. The result aligns with our finding that there is less TWS in Sunflower and Leflore Counties. Furthermore, our results are in agreement with [22], of which they claimed that there is a decrease in groundwater level in the western edge of the Mississippi Delta in the cool-season (October to April) which is the result of recharge from the Mississippi River.

Figure 3, the predicted GRACE mascon of the MAP region, shows that the low water level can be seen in the southeast of Arkansas, central parts of Delta in Mississippi, and northeast of Louisiana, in the MAP region. According to [9], there was a significant decline in groundwater levels from 2004–2014 in the Boeuf-Tensas basin. According to National Land Cover Database (NLCD) 2021, more than 50% of the MAP and the Mississippi Delta regions are cultivated. In both regions, the dominant crop types are soybean, cotton, corn, and rice. The greatest volume of water for irrigation is being used for rice, corn, soybean, and cotton, respectively. Based on Louisiana parish reports [42,43,44], in most of the parishes, the groundwater withdrawal for irrigating rice and other crops has increased over time.

Ref. [45] observed a large disagreement in GRACE-driven data and groundwater model output in the Mississippi Embayment aquifer where the modeled groundwater storage decline was ~4 times greater than GRACE data estimates. They also compared the groundwater storage changes from GRACE with the groundwater level monitoring data, which were in good agreement in most aquifers, and is suggesting that GRACE is capturing groundwater dynamics successfully.

In the Mississippi Embayment aquifer, the irrigation water, which is mostly from groundwater (~84–88%), is equal to or even 50% more than the amount being used in the California Central Valley [2]. Based on these withdrawal estimates, it is expected that TWS shows a great reduction as regional groundwater models (Mississippi Embayment Regional Aquifer System (MERAS) model) suggests (~−120 km^3^ over the 15 year GRACE period), which is not similar to [2] results.

Supported in our results is RFM can be a proper and accurate method to downscale the GRACE mascon images. Ref. [13] is further evidenced by the claim that RFM was the best model out of the four models investigated to predict GRACE values. Their work [13] reported a 0.83 correlation coefficient value.

## 5. Conclusions

Accurately detecting groundwater storage is critical for water management purposes and for achieving this goal, accessing accurate data is crucial. MRVA is a relatively large aquifer in Mississippi and there is evidence and complaints about water decreasing during these past years. We tried to use the GRACE mascon image, which is supposed to show the groundwater level, to see how the GRACE mascon water level is accurate for small areas such as MAP and the Mississippi Delta. If water level changes could be seen in GRACE mascon and align with in situ data and existing complaints, then water resources managers can benefit from it.

Based on [46], TWS changes could be detected monthly in 300,000 km^2^ or larger regions, and seasonal and annual changes could be calculated for 200,000 km^2^ or larger regions. The coarse resolution of GRACE products restricts its usefulness primarily to regional and global-scale investigations and greatly limits its effectiveness for local-scale studies. However, in the past few years, researchers tried different methods to downscale GRACE products and make them useful on a local scale. We also made an effort to downscale GRACE mascon for a small region such as MAP to make the GRACE products more beneficial for local use. Similar to our study, ref. [17] use GRACE to identify spatiotemporal groundwater trends in Flint River Basin, Georgia. They had satisfying results to show the monthly water level anomalies in 5 km resolution after downscaling by using boosted regression tree model. In another recent study, ref. [47] use random forest algorithm to downscale GRACE mascon for Western Anatolian Basin, Turkey. Their result showed more than 98% correlation between GRACE mascon and downscaled products. Ref. [48] is another study that downscales GRACE products for a relatively small area (Shiyang River Basin, China). Their result has a satisfactory correlation coefficient of over 0.60 to predict the groundwater changes during the period of 2003 to 2019. Hence, there is an increasing need for research efforts focused on downscaling GRACE products and making them applicable to smaller regions. This has become a significant and viable area of study.

In this research, we downscaled the GRACE mascon TWS from 0.5° to ~5 km in MAP and the Mississippi Delta regions by using RFM. The result showed that the TWS is low in the central Mississippi Delta, southeast of Arkansas, and northeast of Louisiana. We confirmed the low groundwater level in the central Delta region as reported by [8] for 2020 that it could be observed using the higher resolution GRACE mascon output from this research. Furthermore, there are reports about the decline of both groundwater and surface water resources in southwest Arkansas and northeast Louisiana [9]. Although in this research we cannot confirm the increase or decrease of water level in the study area, since we downscaled only the month of April GRACE mascon image. Still, the downscaled GRACE mascon image can successfully show the water level in our study area. It is important to highlight that the presence of leakage in GRACE data, particularly in small areas such as MAP, which are in close proximity to the ocean, can significantly contribute to errors in water level estimation.

For future work, we will compare the use of RFM on a time series dataset to determine the changes in water level during time and see how accurate mascon data could be in detecting water depletion in small areas. We also will detect surface water in the area and use that data to accurately calculate the groundwater in the area to use in the RFM for downscaling mascon.

## Figures and Tables

**Table 1:** Variables and their spatial resolutions.

Variables	Source	Spatial Resolution
GRACE mascon	JPL	~56 km
Temperature	PRISM	4 km
Precipitation	PRISM	4 km
Soil Type	USDA	Vector data
Aquifer Thickness	USGS	1 km
DEM	MARIS	10 m
Evapotranspiration	MODIS	500 m
Land Cover	MODIS	1 km
NDVI	MODIS	1 km
Ground-based measurement	YMD Joint Water Management District	Table—point data

**Table 2:** The variable of importance for MAP region.

Variables	Importance
Mean Temperature	0.3552
Precipitation	0.3332
Elevation	0.1550
Aquifer thickness	0.1190
Evapotranspiration	0.0179
NDVI	0.0086
Soil Type	0.0055
Land Cover	0.0045

**Table 3:** Statistical metrics of the RFM for MAP region.

	Training Data	Validating Data
R-Squared	0.88	0.85
Root Mean Squared Error	4.9	5.36
Mean Absolute Error	2.49	2.66

## Data Availability

Not applicable.

## Abbreviations

BRT	boosted regression tree
CART	classification and regression trees
DEM	digital elevation model
FO	Follow-on
GAM	Generalized Additive Model
GRACE	Gravity Recovery and Climate Experiment
GWL	Groundwater Level
GWLA	Groundwater Level Anomalies
LMRAA	lower Mississippi River alluvial aquifer
LST	land surface temperature
MAP	Mississippi Alluvial Plain
MASCON	mass concentration pixel
MERAS	Mississippi Embayment Regional Aquifer System
MODIS	Moderate Resolution Imaging Spectroradiometer
NDVI	Normalized difference vegetation index
NLCD	National Land Cover Database
NRCS	Natural Resources Conservation Service
NSE	Nash-Sutcliffe efficiency
PRISM	Parameter-elevation Regressions on Independent Slopes Model
RFM	Random Forest Model
RMSE	Root-mean Square Error
R^2^	correlation coefficient squared
TWS	Total Water Storage
TWSA	Total Water Storage Anomalies
USDA	United States Department of Agriculture
VIMP	Variable Importance Measures Predictive
VI	Variables of Importance
YMD	Yazoo Mississippi Delta Join Water Management District

## Appendix A

**Table A1:** An example of the first 10 rows of the input table in RFM in ArcGIS Pro.

OID	Precipitation	Temperature	Aquifer Thickness	Elevation	NDVI	Land Cover	Soil Type	Mascon	Evapotranspiration
10	159.201	15.0515	159.4386	51	0.3148	0	<Null>	18.50835	25,407
12	137.692	15.209	129.2807	59	0.6766	4	ML	18.50835	9539
15	176.948	14.8774	158.6435	59	0.4417	7	CH	18.50835	154
20	152.015	15.167	141.2935	54	0.2993	7	CL	18.50835	152
24	170.43	14.8165	160.3357	60	0.3275	7	CL	18.50835	153
26	188.837	15.0375	155.9749	55	0.2672	7	CH	18.50835	143
40	179.737	14.8369	115.5124	60	0.5254	7	CH	18.50835	152
44	142.989	15.326	118.3262	68	0.5163	4	ML	18.50835	28,415
46	184.275	14.8024	163.988	56	0.3935	7	CH	18.50835	149
50	180.353	14.7365	110.0095	56	0.4169	7	CL	18.50835	155
